# Prenatal androgen-receptor activity has organizational morphological effects in mice

**DOI:** 10.1371/journal.pone.0188752

**Published:** 2017-11-27

**Authors:** Sabine E. Huber, Bernd Lenz, Johannes Kornhuber, Christian P. Müller

**Affiliations:** Department of Psychiatry and Psychotherapy, University Clinic, Friedrich-Alexander University Erlangen-Nürnberg (FAU), Erlangen, Germany; Radboud University Medical Centre, NETHERLANDS

## Abstract

Prenatal sex hormones exert organizational effects. It has been suggested that prenatal sex hormones affect adult morphological parameters, such as the finger length. Especially the second-to-fourth finger length (2D:4D) ratio has been implicated to be modified when exposed to higher androgen levels *in utero*. Here we show in a mouse model that experimental manipulation of the prenatal androgen level, by blocking the androgen receptor with flutamide or activating the androgen receptor with dihydrotestosterone (DHT), leads to changes in the length of the fingers of all paws in males and females. In addition to that, also total paw length and the 2D:4D ratio was affected. In males treated with DHT, the 2D:4D ratio was increased, while flutamide-treatment in females led to a reduced 2D:4D ratio. We also measured other parameters, such as head size, body length and tail length and demonstrate that body morphology is affected by prenatal androgen exposure with more prominent effects in females. Another factor that is thought to be influenced by early androgens is handedness. We tested mice for handedness, but did not find a significant effect of the prenatal treatment. These findings demonstrate that prenatal androgen activity is involved in the development of body morphology and might be a useful marker for prenatal androgen exposure.

## Introduction

The constitution of the brain and an individual’s behavior are strongly influenced by sex hormones [1–6]. Sex hormones comprise androgens, estrogens and progestogens. Based on their exposure time prenatal and postnatal sex hormone effects can be distinguished [7, 8]. Sex hormones are produced throughout the whole life. However, there are strong surges during prenatal development and puberty [8], which are critical periods for the sexual differentiation of the brain [6, 9–10]. Testosterone is the major male sex hormone and the dominant androgen. It controls the masculinization of the genital and the brain [6, 11]. Dihydrotestosterone (DHT) is the main metabolite of testosterone. It is metabolized from testosterone by the enzyme 5-alpha-reductase [12]. Both testosterone and DHT bind to the androgen receptor (AR) and activate it. However, DHT has a higher affinity to the receptor than testosterone [12]. Antiandrogens or AR antagonists block the receptor and prevent androgens from expressing their function. A selective antagonist of the AR is flutamide (Flu), which is a synthetic non-steroidal antiandrogen. It has no androgenic, estrogenic, antiestrogenic or progestogenic activity [13, 14]. It acts by blocking the AR sites and prevents translocation of the hormone receptor complex to the nucleus [15]. Flutamide also crosses the placental barrier [13, 16].

Sex hormones can exert their effect on the developing brain by organizational and activational effects [1, 2, 4, 17]. The organizational-activational hypothesis states that organizational effects of sex hormones lead to irreversible structural and functional changes of the body and the brain which persist throughout life [1–3, 18]. Organizational effects occur during critical pre- and postnatal periods and in early development [4, 17–19].

Two factors that are thought to be influenced by sex hormone exposure early in life are handedness and the second-to-fourth digit (2D:4D) ratio. Handedness is a marker of cerebral lateralization and an expression of brain asymmetry [1, 20]. There is experimental evidence showing that cerebral lateralization is not an exclusive human trait [21]. Mice also exhibit a paw preference [20, 21].

The hormonal milieu in utero has been associated with finger length in humans [22] and in rodents [6, 14, 23–26]. A widely acknowledged biomarker for prenatal exposure to sex hormones is the 2D:4D ratio [1, 22, 27–28]. The 2D:4D ratio is the length ratio of the second digit (2D, index finger) to the fourth digit (4D, ring finger). The 2D:4D ratio is sexually dimorphic [22, 29]. Males usually have longer 4D than 2D. In females, 2D is usually of equal or same length than 4D [14, 22]. The 2D:4D ratio is, therefore, smaller (2D:4D < 1) in men than in women (2D:4D ≥ 1). A multitude of correlation studies have been performed using this marker and found correlations to e.g. left-handedness [30], aggression [31], and alcohol-addiction [32]. However, this marker has not been experimentally validated so far, even though it was shown that prenatal hormonal treatment influences adult finger length in mice [14]. High exposure to androgens during embryonic development is associated with a lower 2D:4D ratio [22]. An experimental study in mice showed that artificially increased higher levels of DHT during the finger cartilage development led to a decreased 2D:4D ratio in CD 1 mice [14]. In Wistar rats, the length of 2D, 4D, and the 2D:4D ratio were influenced by elevated levels of maternal testosterone. The 2D:4D ratio was masculinized [6]. A sexual dimorphism was found for left front paws, 2D and 4D, as well as for right front paw 2D. All digits were shorter in females [6].

Here we asked how androgens in the embryonic development influence the 2D:4D ratio and also other morphological features, such as head size, body length and tail length in adult mice.

## Material and methods

### Animals

Polygamous breeding pairs (2♀:1♂) were created with 10 week old male and 8 week old female CD 1 mice (Charles River Sulzfeld, Germany). Mice were group-housed in standard macrolon cages with food and water available *ad libitum*. They were kept in a 12:12 hour light/dark cycle (lights on at 7 am). Room temperature was maintained between 19°C and 22°C at a humidity of 55% (±10%). All experiments were carried out in accordance with the National Institutes of Health guidelines for the humane treatment of animals and the European Communities Council Directive (86/609/EEC) and approved by the Committee on the Ethics of Animal Experiments of the Government of Mittelfranken.

### Prenatal treatment

Dam mice were checked daily for a vaginal plug, occurrence of a plug was taken as embryonic day 0.5 (E0.5). The daily prenatal treatment consisted of two intraperitoneal (i.p.) injections on the days E12.5 –E15.5 [14] spaced 30 minutes apart. Male and female offspring of CD 1 dam mice were prenatally treated with four different treatments: (1) vehicle (corn oil (Sigma Aldrich, Taufkirchen, Germany) with 1% ethanol)–vehicle, (2) flutamide (120 mg/kg, Sigma Aldrich, Taufkirchen, Germany)–DHT (2 mg/kg, Sigma Aldrich, Taufkirchen, Germany), (3) vehicle—DHT (4) flutamide—vehicle. The AR antagonist flutamide [13] and the active metabolite of testosterone, DHT, were dissolved in vehicle. Based on the study of Zheng & Cohn [14] we used DHT for prenatal AR activation, and the doses used for flutamide and DHT were also chosen based on [14]. Pups were counted after birth, but otherwise left undisturbed until weaning and gender determination at 3–4 weeks of age and start of experiments. In total 70 male offspring (Veh-Veh n = 19; Flu-DHT n = 22; Veh-DHT n = 10; Flu-Veh n = 19) and 62 female offspring (Veh-Veh n = 14; Flu-DHT n = 17; Veh-DHT n = 13; Flu-Veh n = 18) were used for the experiments. Morphological assessment was performed at an age of approximately 8 weeks. In order to evaluate sex specific effects of hormones, male and female offspring were tested and analyzed separately.

### Digit measurements

**Fig 1:**
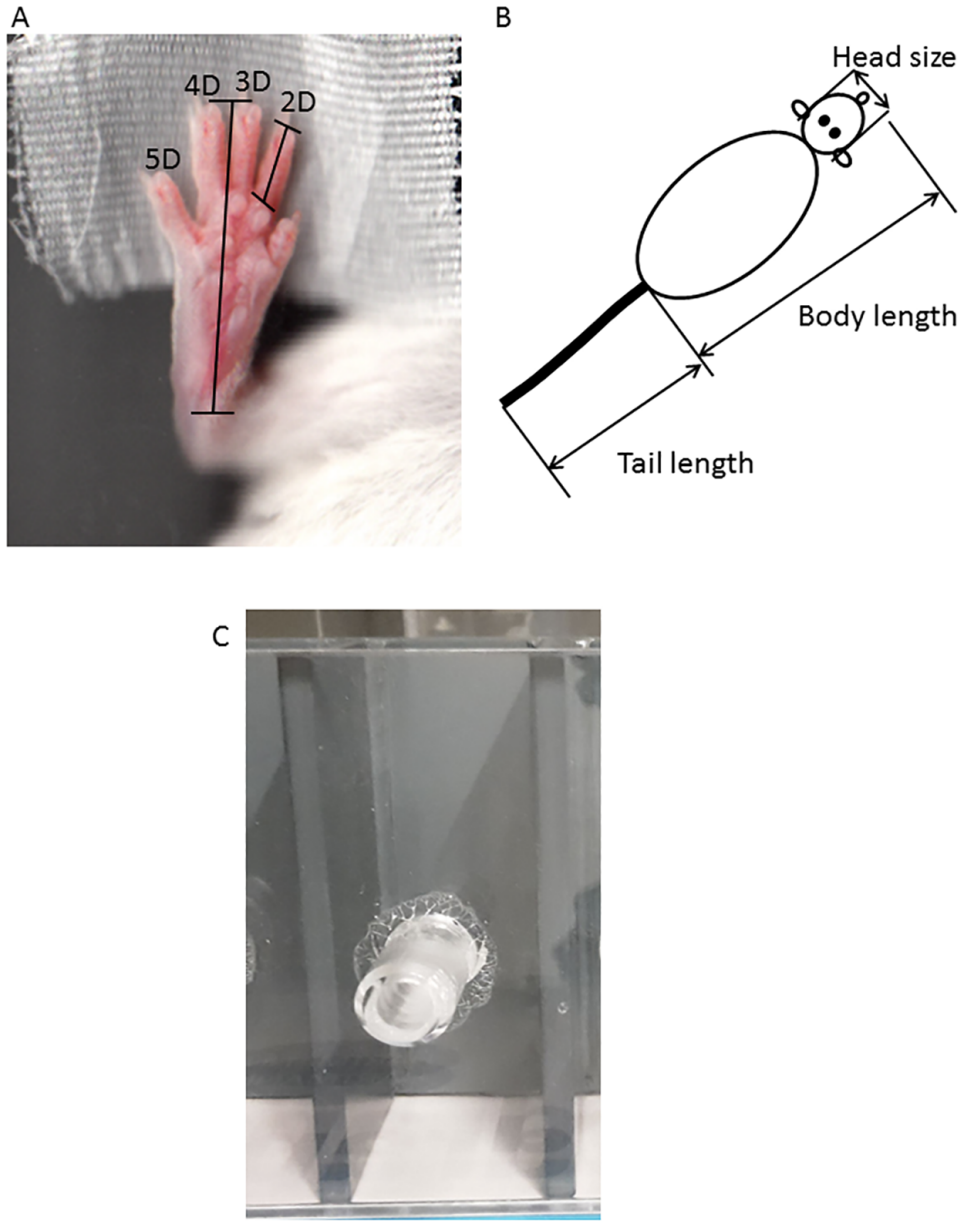
Morphological measurements and handedness box. (A) Scan of a fixed paw with schematic illustration of digit measurement. (B) Sketch of mouse with illustration of morphological parameters. (C) Picture of a compartment of the handedness box. Mice were placed in the box and reaches with one paw through the tubing to obtain a food pellet were counted.

Prenatally treated mice under short isoflurane anesthesia were placed on a Scanner (CanoScan Lide 210, Krefeld, Germany) and scans of the tape fixed paws were made (n = 10-22/group). To measure the paw and digit length, an observer blind to the treatment analyzed the scans using GIMP (GNU Image Manipulation Program). The length of each digit (thumb excluded) and of each paw (left and right, as well as front and hind) was measured. A digit measurement was taken from the basal crease to the tip of the finger, with the nail being excluded (Fig 1A). The measurements were then used to calculate the 2D:4D ratio for the mice.

### Body measurement

Head size was measured using a digital caliper (Hammacher Instrumente Solingen, Germany), while body and tail length were measured with a ruler while mice were under anesthesia (n = 4-14/group). Head size was measured as the distance from ear to ear. Body length was measured from the nose to the beginning of the tail. Tail was measured from the beginning to the tip (Fig 1B).

### Handedness

Handedness is a proxy for lateralization of the brain [1, 33, 34]. To test if the prenatal treatment had an effect on lateralization, we tested for handedness in prenatally treated mice (n = 10-25/group). The apparatus for the handedness consisted of a Plexiglas box with grey walls and a see-through front. There were five compartments next to each other with one compartment being 11.5 cm high, 3.8 cm wide and 5.5 cm deep. A 3 cm long feeding tube with a diameter of 0.9 cm was placed in the middle of the clear front (5.75 cm high and 1.45 cm middle, Fig 1C, manufactured in house) [35–37]. Animals were food deprived for approximately 24 hours before testing and primed with the test food (TSE dustless precision pellets). On the test day, animals were placed into a compartment for 20 min. 25 TSE pellets were placed within reach in the feeding tubes. The compartment was cleaned between subjects with 1% acetic acid to avoid any olfactory cues influencing behaviors. Paw reaches into the feeding tube were observed and counted. A total of 50 paw reaches was observed. An exclusion criterion was when less than 50 reaches within 20 min were executed. Animals were classified as left-handed, right-handed or ambidextrous based on the number of reaches they executed with the right paw. Mice classified as left-handed executed 0–17 right paw reaches, while mice classified as right-handed executed 33–50 right paw reaches. Animals reaching between 18–32 times with the right paw were classified as ambidextrous.

### Statistics

All quantitative data were expressed as mean ± Standard error of the mean (SEM). Data were analyzed using two-way ANOVAs followed by pre-planned comparisons [38] using Fishers’s LSD tests with Bonferroni-correction when appropriate. A p-value of <0.05 was considered statistically significant. Data were analyzed using IBM SPSS statistics Version 21 for Windows (SPSS Inc., Chicago, IL, USA).

## Results

### Prenatal sex hormones shape adult finger and paw length in males

**Fig 2:**
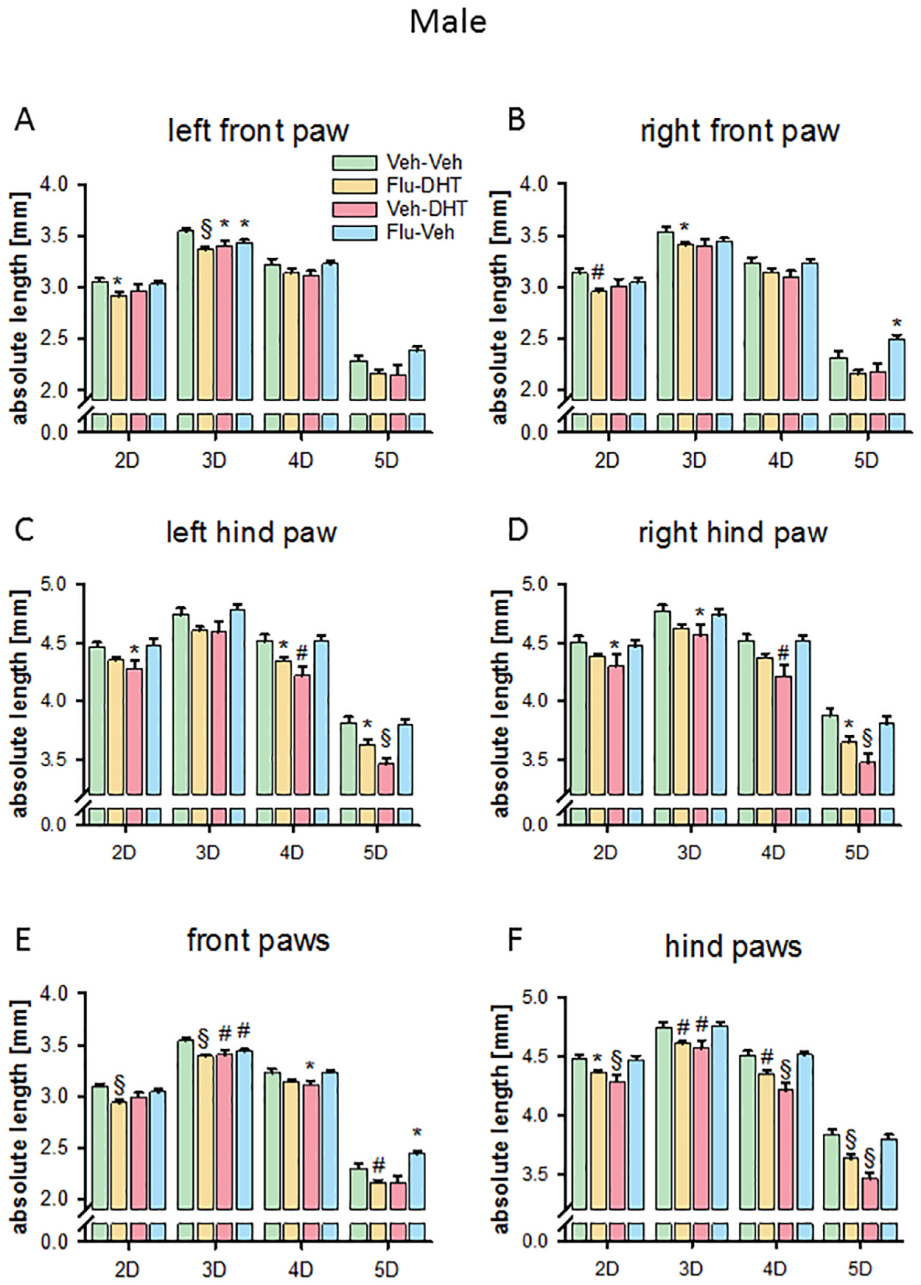
Absolute finger length is altered by prenatal treatment in male mice. Length of digits on the left (A) and the right (B) front paw. Length of digits on the left (C) and the right (D) hind paw. (E) Digit length of pooled (left + right) front paws. (F) Digit length of pooled (left + right) hind paws. Data represented as mean ± Standard error of the mean (SEM). * p<0.05, # p<0.01 and § p<0.001 compared to control. Veh—Vehicle; Flu—Flutamide; DHT—Dihydrotestosterone.

The single digit length was changed in both the front and hind paws of prenatally treated adult males. Prenatal treatment with Flu-Veh resulted in a shorter 3D (p<0.05; Fig 2A) on the left front paw in adult males, while the treatment did not affect length of the digits 2, 4 and 5. Prenatally Veh-DHT-treated males had a reduced 3D length on the left front paw (p<0.05; Fig 2A), while the digits 2, 4 and 5 remained unaltered. However, prenatal treatment with Flu-DHT resulted in shorter 2D, as well as 3D of adult males on the left front paw (p<0.05; Fig 2A), while 4D and 5D were not changed. In the right front paw prenatal treatment with Flu-Veh increased the length of 5D (p<0.05; Fig 2B) in adult males. Prenatal treatment with Veh-DHT had no effect on right front paw digit length in adult males, while prenatal Flu-DHT treatment resulted in shorter 2D and 3D (p<0.05; Fig 2B) in adult males.

In the left hind paw, no effect of prenatal Flu-Veh treatment was observed for single digit length. Prenatal treatment with Veh-DHT, however, led to shorter 2D, 4D and 5D in adult males (p<0.05; Fig 2C). Pre-treatment with Flu, in the Flu-DHT group, also reduced the length of 4D and 5D in adult males on the left hind paw (p<0.05; Fig 2C). On the right hind paw, prenatal treatment with Flu-Veh had no effect on finger length, however, all measured fingers (2D-5D) were shorter in adult males prenatally treated with Veh-DHT (p<0.05; Fig 2D). The pre-treatment with Flu, in the Flu-DHT-treated group, resulted in shorter 5D (p<0.05; Fig 2D).

Because of the similar pattern in right and left paw, we pooled the data for analysis to enhance power. We found a shorter 3D and longer 5D in prenatally Flu-Veh-treated males (p<0.05; Fig 2E) on the front paws. In the prenatally Veh-DHT-treated males front paw 3D and 4D were shorter (p<0.05; Fig 2E). In adult males treated prenatally with Flu-DHT, the length of 2D, 3D and 5D were reduced on front paws (p<0.05; Fig 2E). No effect of prenatal Flu-Veh-treatment was found on adult male hind paws, but treatment with Veh-DHT resulted in shorter 2D, 3D, 4D and 5D (p<0.05; Fig 2F). Furthermore, pre-treatment with Flu, in the Flu-DHT males also reduced the length of all four digits (2D-5D, p<0.05; Fig 2F) on hind paws.

**Fig 3:**
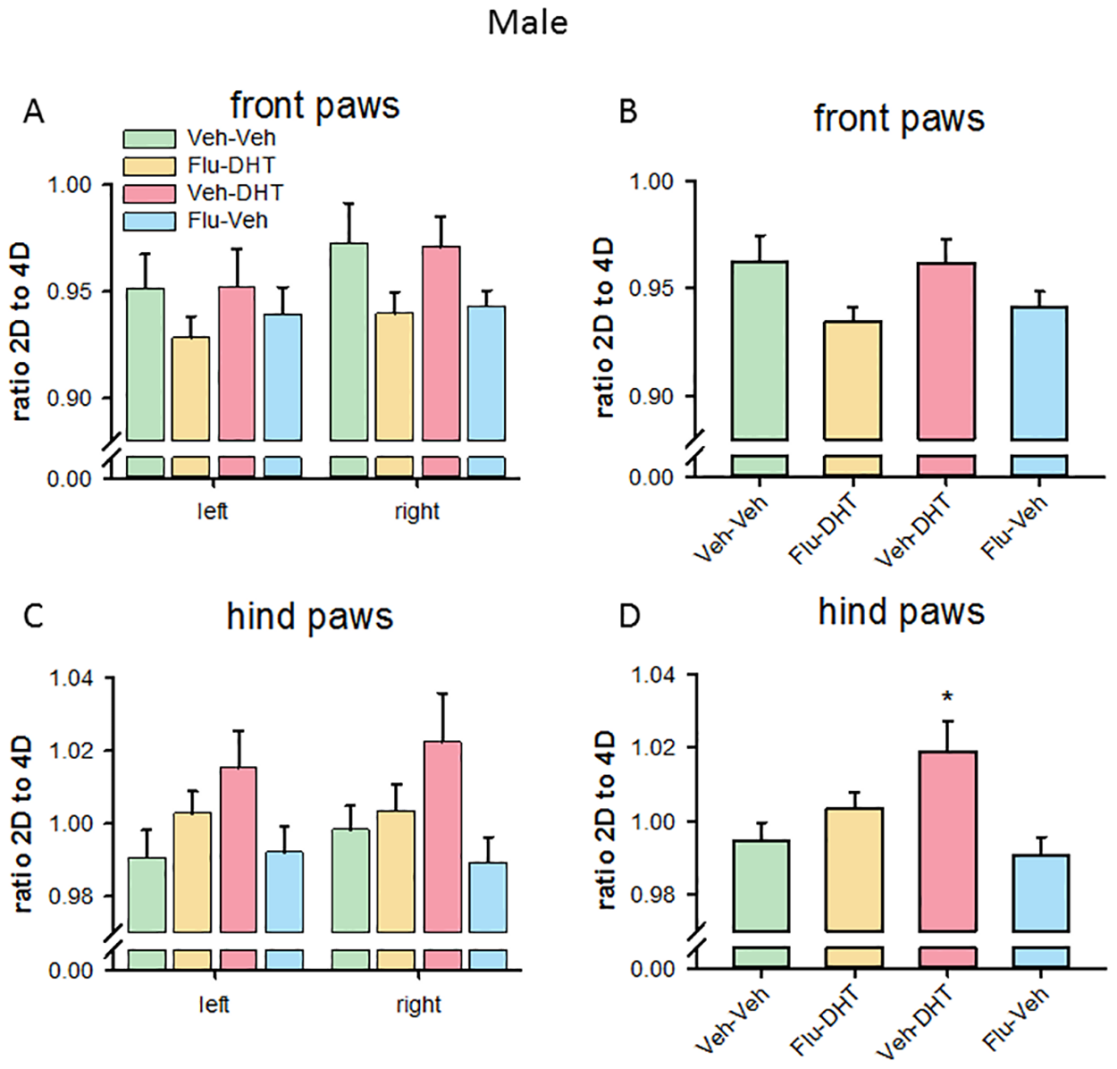
Increased 2^nd^ to 4^th^ digit (2D:4D) ratio in hind paws of Vehicle-Dihydrotestosterone-treated males. (A) 2D:4D ratio on the left and right front paw. (B) 2D:4D ratio of the pooled (left + right) front paws. (C) 2D:4D ratio on the left and right hind paw. (D) 2D:4D ratio of the pooled (left + right) hind paws. Data represented as mean ± Standard error of the mean (SEM). * p<0.05 compared to control. Veh—Vehicle; Flu—Flutamide; DHT—Dihydrotestosterone.

The analysis of the 2D:4D ratio of the front paws in males, showed no effect of prenatal treatment on either left (F_(3,66)_ = 0.708, p = 0.550; Fig 3A) or right (F_(3,66)_ = 1.801, p = 0.156) front paw. Pooling the paws, we found no statistically significant effect of prenatal treatment (F_(3,136)_ = 2.326, p = 0.078; Fig 3B) for the 2D:4D ratio. The analysis of the 2D:4D ratio of the hind paws in males, found no effect of prenatal treatment on either left (F_(3,66)_ = 1.801, p = 0.156, Fig 3C) or right (F_(3,66)_ = 2.573, p = 0.061) hind paw. However, pooling the hind paws, we found a prenatal treatment effect (F_(3,136)_ = 4.231, p = 0.007). In this analysis, prenatal Veh-DHT treatment increased the 2D:4D ratio in males (p<0.05, Fig 3D). Altogether this suggests that prenatal AR activation influences the length of single digits and enhances the 2D:4D ratio in the hind paws of adult male mice.

**Fig 4:**
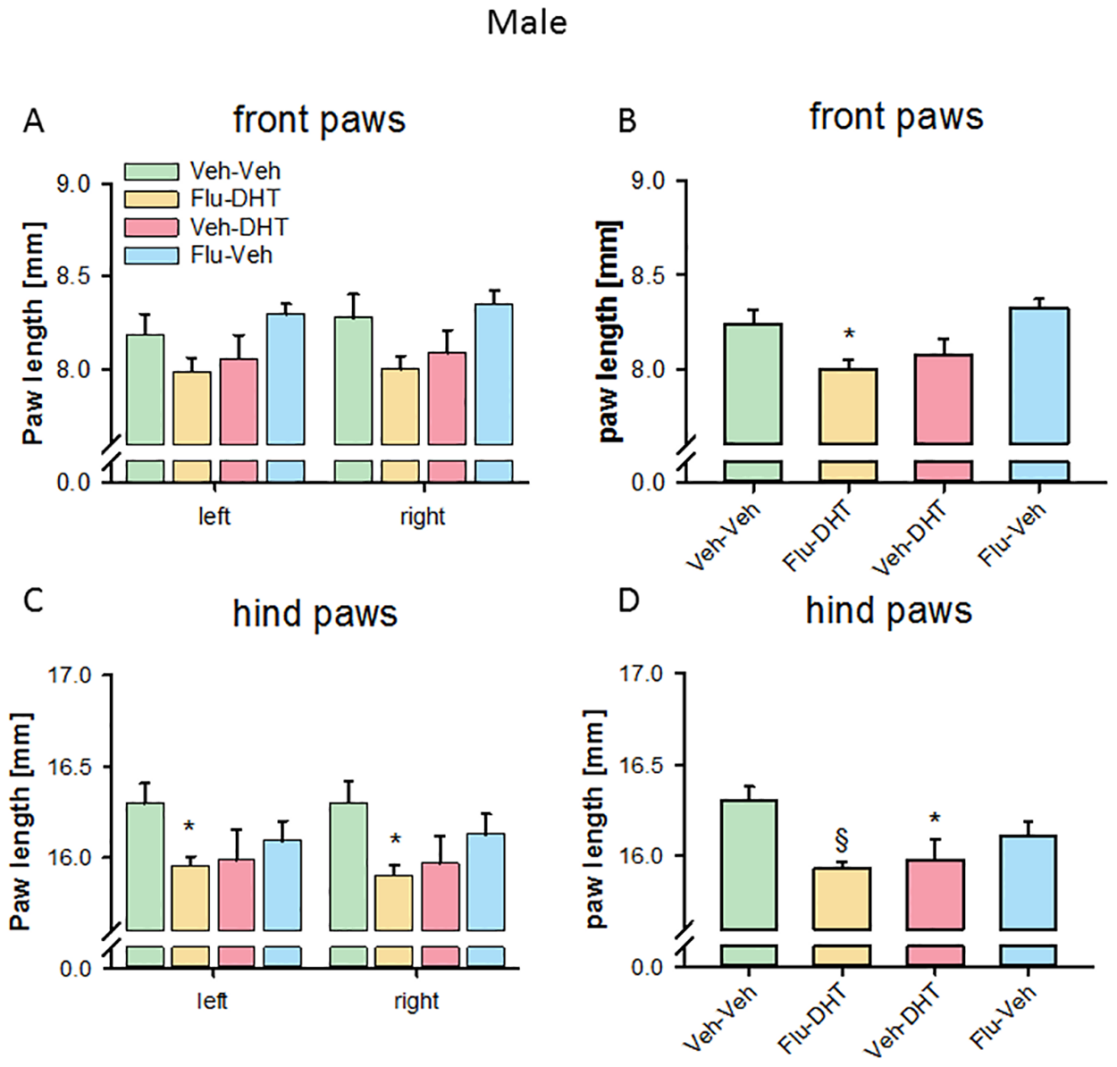
Shorter paws in males prenatally treated with Flutamide-Dihydrotestosterone. (A) Paw length of the left and right front paw. (B) Paw length of pooled (left + right) front paws. (C) Paw length of the left and right hind paw. (D) Paw length of pooled (left + right) hind paws. Data represented as mean ± Standard error of the mean (SEM). * p<0.05 and § p<0.001 compared to control. Veh—Vehicle; Flu—Flutamide; DHT—Dihydrotestosterone.

Prenatal treatment also affected adult paw length. We found an overall prenatal treatment effect on right front paw length (F_(3,66)_ = 3.012, p = 0.036; Fig 4A), but only a trend on left front paw length (F_(3,66)_ = 2.684, p = 0.054) in males. However, pre-planned comparisons showed no significant between-group differences. Pooling the right and left paw, we found an effect of prenatal treatment (F_(3,136)_ = 5.801, p = 0.001) on the male front paw length. The prenatally Flu-DHT-treated males had shorter front paws than controls (p<0.05; Fig 4B).

Analyzing the paw length in hind paws of males, we found no effect of prenatal treatment in the left (F_(3,66)_ = 2.482, p = 0.069), but in the right (F_(3,66)_ = 3.327, p = 0.025) hind paw. Pre-planned comparisons revealed a shorter paw length in prenatally Flu-DHT-treated males both in the left and right hind paws (p<0.05; Fig 4C). Pooling the left and right paw, this effect was strengthened. We found an effect of prenatal treatment (F_(3,136)_ = 5.898, p = 0.001) for pooled hind paw length. Both the Veh-DHT-treated and the Flu-DHT-treated males had smaller paws (p<0.05; Fig 4D). Overall this suggests that prenatal AR activation reduces paw length in male mice.

### Prenatal sex hormones affect finger and paw length in adult females

**Fig 5:**
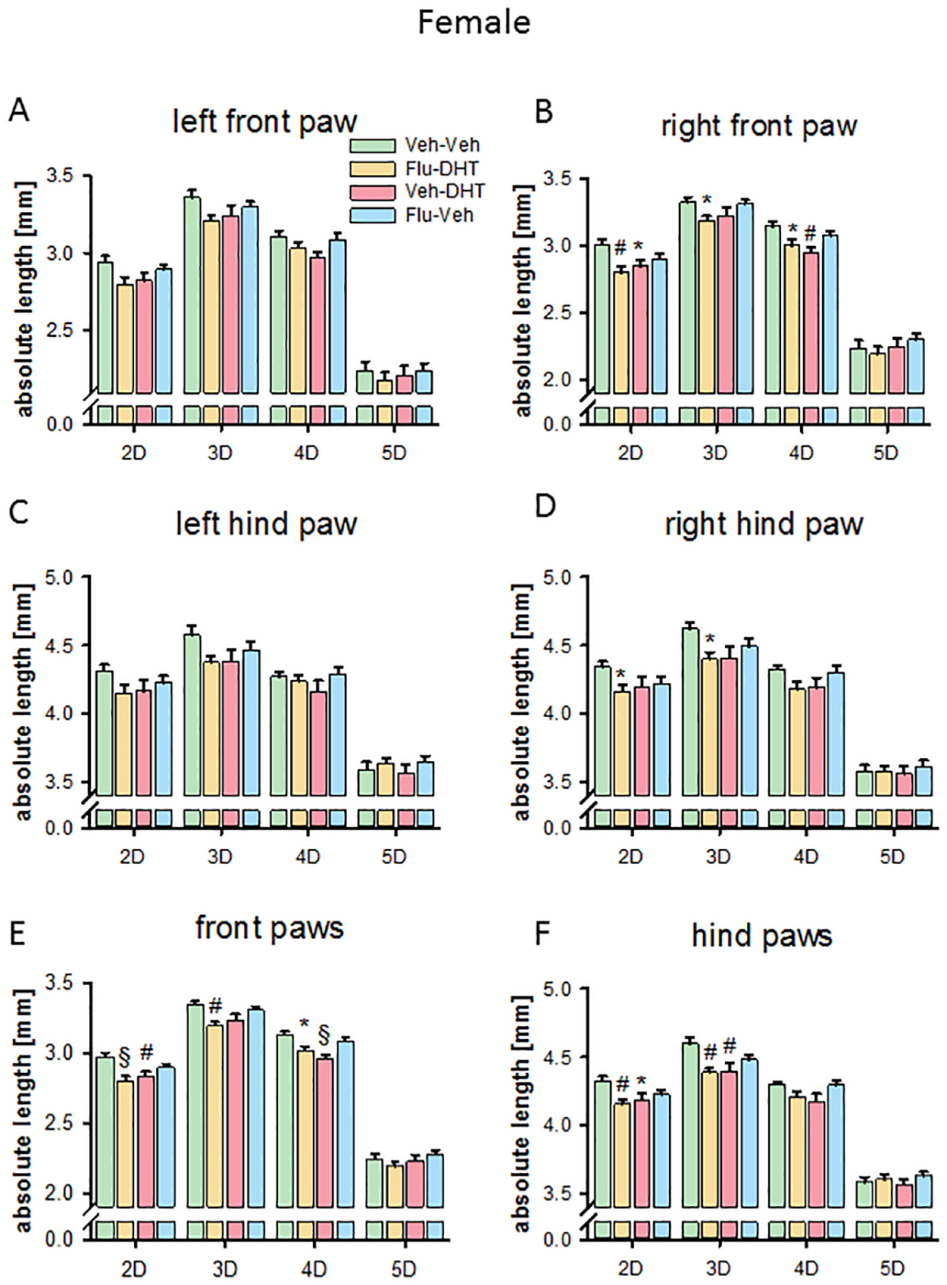
Absolute length of fingers reduced by prenatal treatment in females. Length of digits on the left (A) and the right (B) front paw. Length of digits on the left (C) and the right (D) hind paw. (E) Digit length of pooled (left + right) front paws. (F) Digit length of pooled (left + right) hind paws. Data represented as mean ± Standard error of the mean (SEM). * p<0.05, # p<0.01 and § p<0.001 compared to control. Veh—Vehicle; Flu—Flutamide; DHT—Dihydrotestosterone.

Prenatal treatment had a significant influence on single digit length in both front and hind paws in females. The digit length of the left front paw in adult females was not affected by prenatal treatment (Fig 5A). However, we found an effect on the right front paw. Prenatal treatment with Veh-DHT reduced the length of 2D and 4D (p<0.05; Fig 5B) in adult females. Pre-treatment with Flu, in the Flu-DHT-treated females, resulted in shorter 2D, 3D, as well as 4D (p<0.05; Fig 5B) at adult age. On the left hind paw we did not find an effect of prenatal treatment on finger length (Fig 5C). For the right hind paw we found an effect only in the prenatally Flu-DHT-treated females. The length of 2D and 3D were reduced (p<0.05; Fig 5D).

Because of the similar pattern in right and left paw, we pooled the data for analysis to enhance power. We found a shorter 2D and 4D in prenatally Veh-DHT-treated females (p<0.05; Fig 5E) on the front paws. In the prenatally Flu-DHT-treated female front paw, 2D, 3D and 4D were shorter than in controls (p<0.05; Fig 5E). Prenatal treatment with Veh-DHT resulted in shorter 2D and 3D in adult female hind paws (p<0.05; Fig 5F). Furthermore, pre-treatment with Flu, in the Flu-DHT females, also reduced the length of 2D and 3D (p<0.05; Fig 5F) on hind paws.

**Fig 6:**
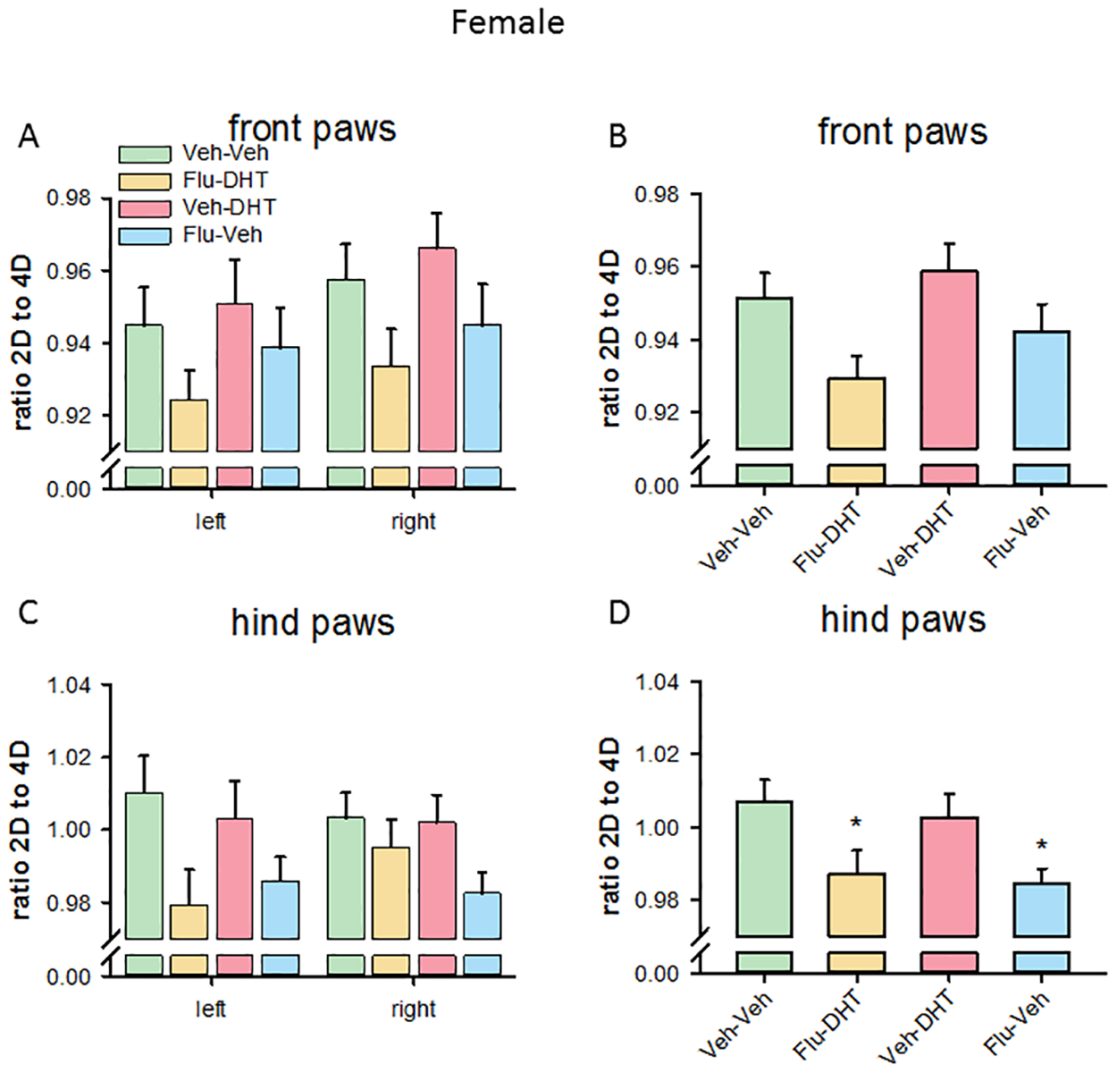
Decreased 2^nd^ to 4^th^ (2D:4D) digit ratio in hind paws of Flutamide-Vehicle-treated females. (A) 2D:4D ratio on the left and right front paw. (B) 2D:4D ratio of the pooled (left + right) front paws. (C) 2D:4D ratio on the left and right hind paw. (D) 2D:4D ratio of the pooled (left + right) hind paws. Data represented as mean ± Standard error of the mean (SEM). * p<0.05 compared to control. Veh—Vehicle; Flu—Flutamide; DHT—Dihydrotestosterone.

The analysis of the 2D:4D ratio of the front paws in females showed no effect of prenatal treatment on either left (F_(3,58)_ = 1.277, p = 0.291; Fig 6A) or right (F_(3,58)_ = 2.066, p = 0.115) front paws. Pooling the right and left front paws, we found a statistically significant effect of prenatal treatment (F_(3,120)_ = 3.305, p = 0.023; Fig 6B) for the 2D:4D ratio. However, pre-planned comparisons did not reach statistical significance for single groups.

The analysis of the 2D:4D ratio of the hind paws in females found an effect of prenatal treatment on left (F_(3,58)_ = 2.762, p = 0.050, Fig 6C), but not on right (F_(3,58)_ = 1.959, p = 0.130) hind paws. However, pre-planned comparisons revealed no significant treatment-effect. Pooling the hind paws, we found a prenatal treatment effect (F_(3,120)_ = 4.232, p = 0.007). Prenatal Flu-Veh and Flu-DHT treatment decreased the 2D:4D ratio in females, when left and right hind paws were pooled (p<0.05, Fig 6D). Altogether this suggests that prenatal AR antagonism reduced the 2D:4D ratio in adult females.

**Fig 7:**
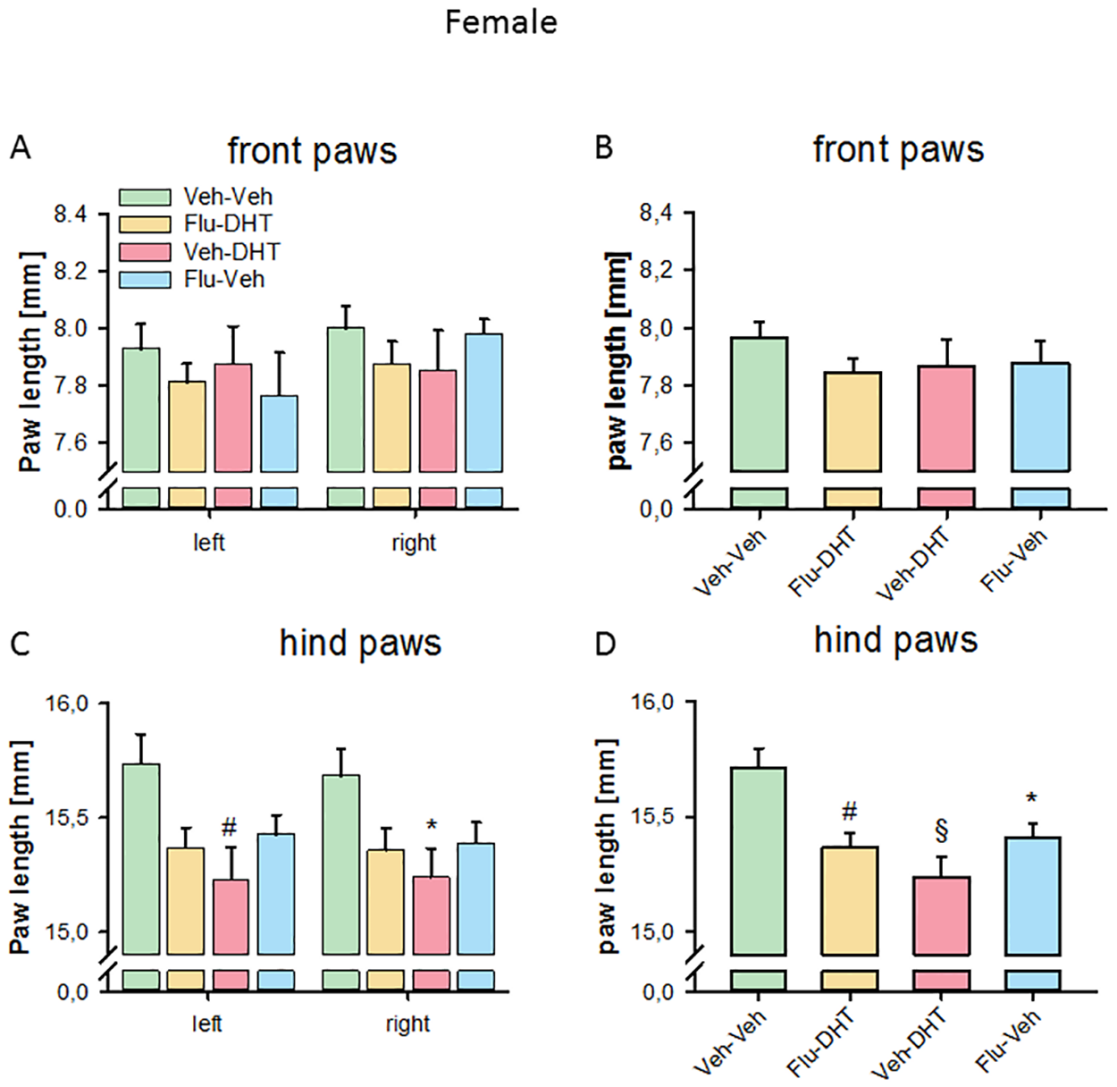
Shorter paws in females prenatally treated with Vehicle-Dihydrotestosterone. (A) Paw length of the left and right front paw. (B) Paw length of pooled (left + right) front paws. (C) Paw length of the left and right hind paw. (D) Paw length of pooled (left + right) hind paws. Data represented as mean ± Standard error of the mean (SEM). * p<0.05, # p<0.01 and § p<0.001 compared to control. Veh—Vehicle; Flu—Flutamide; DHT—Dihydrotestosterone.

We found no prenatal treatment effect on total paw length of the left (F_(3,58)_ = 0.382, p = 0.766) or right (F_(3,58)_ = 0.734, p = 0.536; Fig 7A) front paws in females. Pooling the paws, we also found no effect of prenatal treatment (F_(3,120)_ = 0.520, p = 0.669) on the female front paw length (Fig 7B).

Analyzing the paw length in hind paws of females, we found an effect of prenatal treatment in the left (F_(3,58)_ = 3.549, p = 0.020) and in the right (F_(3,58)_ = 2.904, p = 0.042) hind paw. A shorter paw length was found in prenatally Veh-DHT-treated females both in the left (p<0.01; Fig 7C) and right (p<0.01) hind paw. Pooling the left and right paw, this effect was strengthened. We found an effect of prenatal treatment (F_(3,120)_ = 6.644, p<0.001) for pooled hind paw length. All treatment groups had smaller paws in comparison to controls (Flu-DHT: p<0.01; Veh-DHT: p<0.001 and Flu-Veh: p<0.05; Fig 7D). Overall this suggests that prenatal AR agonism and antagonism reduced paw length in females.

### Prenatal sex hormones had marginal effects on body morphology depending on gender and treatment

**Fig 8:**
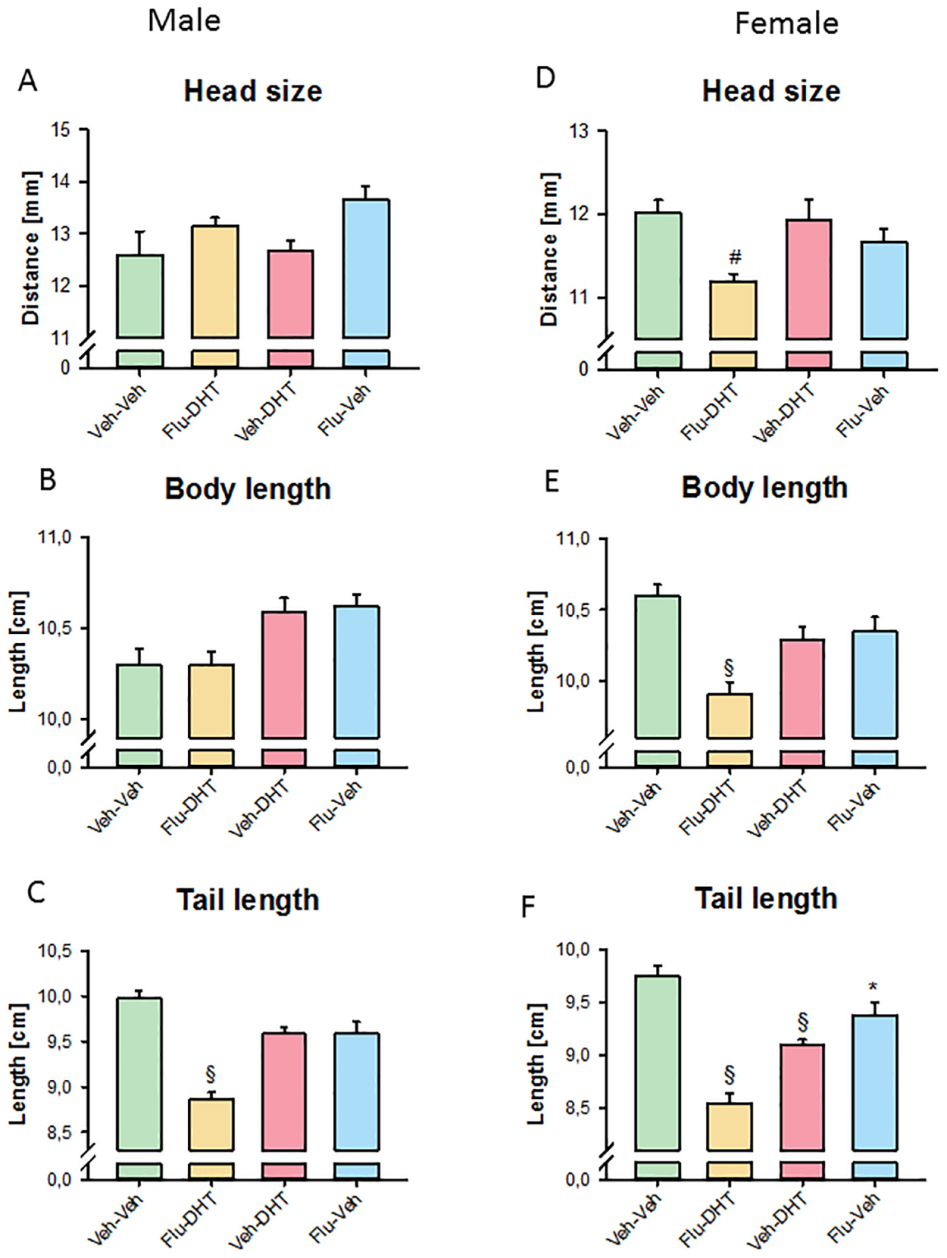
Body morphology in adult prenatally treated males and females. Male (A) head size measured as distance between ears, (B) body length and (C) tail length in cm. Female (D) head size measured as distance between ears, (E) body length and (F) tail length in cm. Data represented as mean ± Standard error of the mean (SEM). * p<0.05, # p<0.01 and § p<0.001 compared to control. Veh—Vehicle; Flu—Flutamide; DHT—Dihydrotestosterone.

We found an effect of prenatal treatment on the head size (F_(3,42)_ = 4.275, p = 0.010; Fig 8A), body length (F_(3,42)_ = 5.247, p = 0.004; Fig 8B) and tail length (F_(3,42)_ = 17.741, p < 0.001; Fig 8C) in adult males. While ANOVA revealed a main effect and pre-planned comparisons were nominally significant, this was no longer apparent after Bonferroni correction, except for shorter tail length in Flu-DHT-treated males. In adult females, we found an effect of prenatal treatment on the head size (F_(3,33)_ = 4.932, p = 0.006; Fig 8D), body length (F_(3,33)_ = 10.315, p < 0.001; Fig 8E) and tail length (F_(3,33)_ = 29.725, p < 0.001; Fig 8F). Prenatally Flu-DHT-treated females displayed smaller measurements for all three parameters (Fig 8D–8F). Furthermore, tail length was shorter in all three treatment groups compared to the control group (Fig 8F).

### No effect of prenatal androgen receptor activity on handedness in adult mice

**Fig 9:**
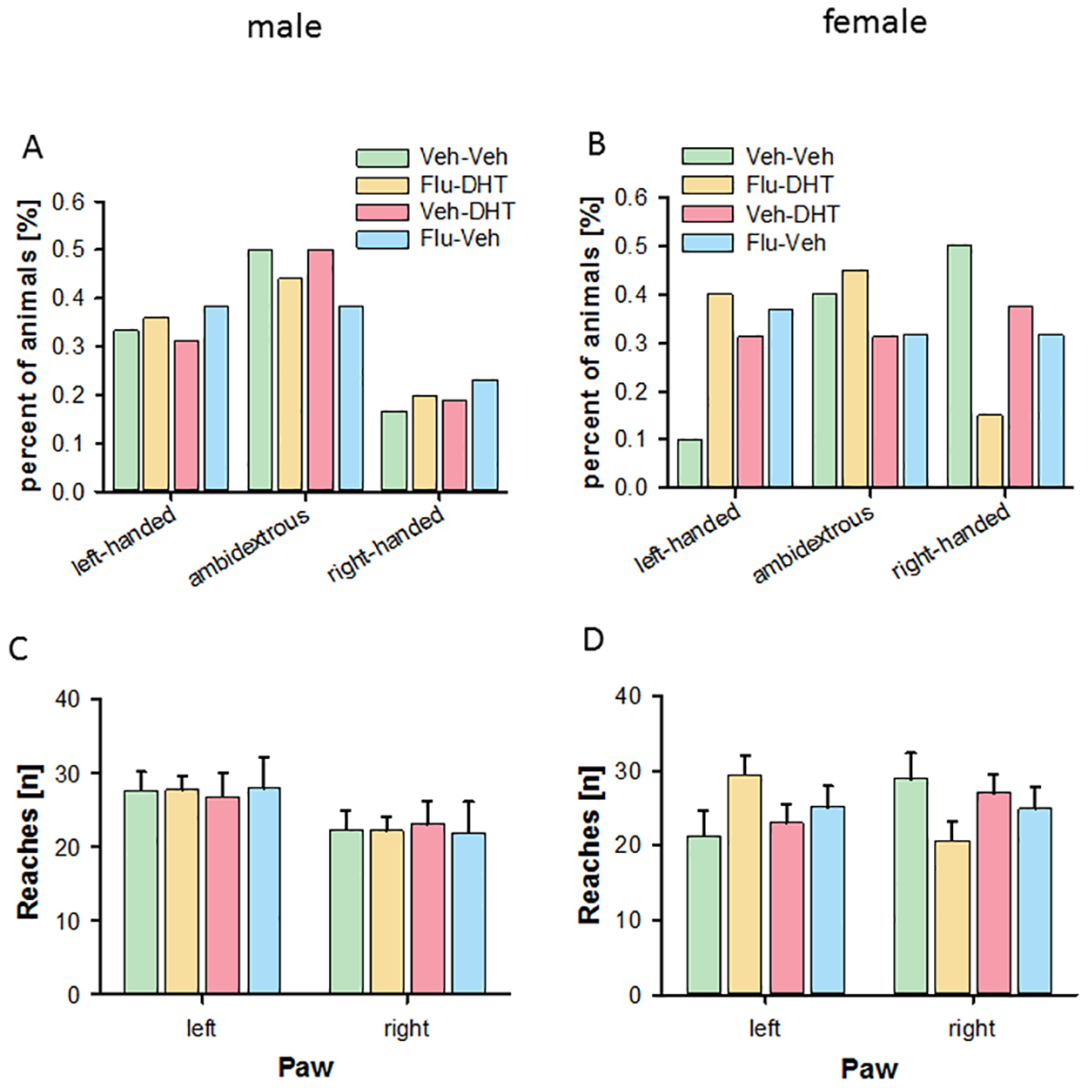
No effect of prenatal treatment on handedness in adult males and females. Percentage of animals preferring left paw, right paw or using both paws equally (ambidextrous) in males (A) and females (B). Mean reaches of paws into feeding tube with each paw in males (C) and females (D). Data represented as mean ± Standard error of the mean (SEM). Veh—Vehicle; Flu—Flutamide; DHT—Dihydrotestosterone.

Examining the percentage of animals classified as left-handed, right-handed or ambidextrous within the groups, we found no major differences between the groups in males (Fig 9A). In the females, we observed that more mice from the control group use the right paw. The Flu-DHT-treated group, by contrast, seemed to prefer the left paw (Fig 9B). The number of animals using both paws equally was high (males: ~40–50%; females: ~30–40%), which might be due to digging behavior that was observed. In males, it seems that the left paw is more preferred at approximately 30% compared to right paw at approximately 20%. Reexamining the right and left paw reaches using the mean paw reaches per group we detected an effect for preferred paw in males (F_(1,136)_ = 6.716, p = 0.011, Fig 9C). Male mice preferred the left paw. However, no differences between prenatal treatment groups were found (p>0.05). In females, there was an interaction effect of prenatal treatment and preferred paw (F_(3,122)_ = 2.690, p = 0.049, Fig 9D). However no differences between treatment groups (F_(3,122)_ = 0.218, p = 0.884) or preferred paw (F_(1,122)_ = 0.139, p = 0.710) were found. In that, in females there was no clear paw preference observed. This data suggests that adult mice display no clear laterality for paw usage and that prenatal AR activity does not change this.

## Discussion

In this study we investigated the role of prenatal AR activation on morphological development in mice. The hind paw 2D:4D ratio was increased in adult males after prenatal AR activation. In females, prenatal AR blockade with either Flu-Veh or Flu-DHT resulted in a decreased 2D:4D ratio. This effect was more pronounced in hind paws compared to front paws. We examined 2D – 5D of the paws and found that nearly all digits were affected by prenatal treatment. However, single digits were affected differently in males and females. Not only digit length, but also paw length was affected by prenatal treatment. Smaller hind paws were found in males after prenatal AR activation in the Veh-DHT or Flu-DHT groups. In females, hind paws of Flu-Veh, Veh-DHT and Flu-DHT-treated mice were smaller. While these results clearly show organizational morphological effects of prenatal ARs, we did not find that higher prenatal androgen levels led to a lower 2D:4D ratio [22]. Several differences in methodology might be responsible for the different findings in our study compared to others [6, 14]. First, the administration route for flutamide was changed from p.o. to i.p. Second, our pregnant dams were injected twice per day with 30 min between injections, while in [14] mice were only injected once daily. The dose, however, was identical to the study of Zheng & Cohn [14]. Third, all gender and treatment-combinations were analyzed here, while a previous study focused on selected groups only [14], e.g. they did not show Flu-treated females or DHT-treated males.

Mouse as well as human studies focusing on the digit ratio frequently differ in their findings [24–26, 30]. In humans, several studies found an inverted U-shaped relationship between digit ratio and behavioral traits, such as mathematical performance or asymmetry [39–43]. Human studies also show sex differences between the digit ratio and behavioral traits. Mostly significant correlations are only found in males [44–47]. This might be due to different sensitive periods of prenatal brain organization. In rodents, sex differences in digit ratios have been inconsistent [48]. Furthermore, digit ratios differ significantly between inbred strains of mice. Strains with higher digit ratios tended to have sex effects in the opposite direction compared to strains with lower digit ratios [24]. These differences may be explained in several ways. There are strain differences in digit ratio as well as in behavior, so the type of mice used might factor into the differences [24]. Another possibility is the sample size of measured animals [24, 48]. Compared to other studies our sample size was considerably lower. Also the actual measurement method for digit length might impact the results [24]. Photographic measurements of 2D:4D ratio in field voles resulted in a higher measurement error and in higher estimates of 2D:4D than X-ray measurements [48, 49]. Also human studies demonstrated the importance of the measurement method. Photocopy based measurements produced lower 2D:4D ratios than direct finger measurements [27, 50]. It was suggested that the digit ratio is influenced by the tissue over the bones and the way the tissue is measured, i.e. the applied pressure while photocopying the hand [27]. The possibility of litter effects might be another explanation for our findings. Depending on the number of males and females in a litter, the endogenous androgen milieu is more pronounced and could overwrite the effect of the exogenous androgens or mask the effects of sex [24]. This is possible because of the way mice develop in the uterine horns of the mother. In rodents, testosterone is transferred between pups in the uterine horn [51, 52]. It was shown that the development and 2D:4D ratio is influenced by neighboring pups [29, 48]. Mice gestating next to males have a larger 2D:4D ratio than those gestating next to females [29]. Animals show a more masculine behavior depending on the place of gestation and the neighbor which they gestate next to [19, 51, 53]. This effect is called the intrauterine position effect (IUP) [29, 51]. It could be shown that females gestating in between two male fetuses, called 2M, exhibit more male like characteristics in sexual behavior, are more aggressive, and have an increased anogenital distance (AGD) [19, 53–56]. However, these effects can be abolished by prenatal flutamide treatment [19, 57]. The placenta produces androgens [58, 59] which might also impact our results. Between days 12–18 of pregnancy, the placenta becomes an important source of androgens in maternal circulation [58, 59]. Pregnant rats show considerable differences in blood levels of androgens [59]. Thus, the differences in endogenous androgens among pregnant females may account for some variation in masculinization of female offspring born to different mothers [58]. Altogether, the present results show that prenatal AR activity changes the growth of digits and paws, thus, influencing also digit ratio. Furthermore, we observed an additive effect of treatment in the prenatally Flu-DHT-treated animals. We can only speculate about this potentiating effect. A possibility could be the involvement of another receptor or pathway that influences the morphological parameters.

In addition to finger and paws, we also measured head, body and tail size. We found that these morphological readouts were also affected by prenatal treatments. A trend for reduced head size, body length as well as tail length was found in females prenatally treated with Flu-DHT. In males, it only reduced tail length. Head size and body length in males displayed a trend to be increased, as well as, tail length in females treated with Flu-Veh prenatally. Overall, this suggests that prenatal activity might be involved in body growth.

Handedness is a marker for brain lateralization [1]. Handedness is supposed to be influenced by early sex hormone exposure [1], thus, representing an organizational effect. Prenatal testosterone promotes right-handedness [1]. Cerebral lateralization is not just a human trait, but was proposed to be also expressed in rodents [21]. However, the degree of lateralization in rodents is unclear. Handedness or paw preference was demonstrated in mice [60, 61]. Female mice display a higher lateralization than male mice [62, 63]. Paw preference also varies among strains as well as substrains of mice [21]. They could show that the preference for the right or left paw is equally distributed, while approximately 20–40% showed no preference. Those animals were termed ambidextrous [64]. We found no effects of prenatal treatment on the paw preference. About 40% of the mice were ambidextrous, like previously postulated. The influence of testosterone on paw preference or the development of laterality is unclear. We selected our time point for prenatal treatment based on the finger ratio development [14]. Therefore, data suggest that handedness and finger ratio are markers for different developmental stages [65, 66]. Thus, the chosen time window for the treatment administration might not influence the development of handedness. An inclusion criterion was determined for paw preference: a mandatory 50 reaches within 20 min. Surprisingly, a rather large number of animals failed this criterion (approx. one third (33%)), possibly because the preferred food pellets were not incentive enough to encourage sufficient reaches into the tube. Altogether, present data suggest that prenatal activation or blockade of the AR did not influence the development of handedness in either male or female mice. However, single digit length in adult males is reduced following prenatal activation of the AR via DHT. Furthermore, activation of the AR also increased the 2D:4D ratio in adult males and reduced the paw length. In adult males, prenatal blockade of the AR with flutamide seems not to influence paw- and finger length. In adult females, single digit length is reduced following prenatal AR activation. However, 2D:4D ratio is reduced following prenatal AR inhibition with flutamide. Paw length in adult females is reduced independent of the type of prenatal treatment. The same applies for the body morphology in females, where specifically tail length is reduced independent of AR activation or blockade.
